# Current Status of Next-Generation Sequencing in Bone Genetic Diseases

**DOI:** 10.3390/ijms241813802

**Published:** 2023-09-07

**Authors:** Natsuko Aida, Akiko Saito, Toshifumi Azuma

**Affiliations:** 1 Department of Biochemistry, Tokyo Dental College, 2-9-18 Kandamisaki-cho, Chiyoda-ku, Tokyo 101-0061, Japan; ayokoyama@tdc.ac.jp (A.S.); tazuma@tdc.ac.jp (T.A.); 2 Oral Health Science Center, Tokyo Dental College, 2-9-18 Kandamisaki-cho, Chiyoda-ku, Tokyo 101-0061, Japan

**Keywords:** next-generation sequencing, genome, bone genetic diseases, genes in bone tissues

## Abstract

The development of next-generation sequencing (NGS) has dramatically increased the speed and volume of genetic analysis. Furthermore, the range of applications of NGS is rapidly expanding to include genome, epigenome (such as DNA methylation), metagenome, and transcriptome analyses (such as RNA sequencing and single-cell RNA sequencing). NGS enables genetic research by offering various sequencing methods as well as combinations of methods. Bone tissue is the most important unit supporting the body and is a reservoir of calcium and phosphate ions, which are important for physical activity. Many genetic diseases affect bone tissues, possibly because metabolic mechanisms in bone tissue are complex. For instance, the presence of specialized immune cells called osteoclasts in the bone tissue, which absorb bone tissue and interact with osteoblasts in complex ways to support normal vital functions. Moreover, the many cell types in bones exhibit cell-specific proteins for their respective activities. Mutations in the genes encoding these proteins cause a variety of genetic disorders. The relationship between age-related bone tissue fragility (also called frailty) and genetic factors has recently attracted attention. Herein, we discuss the use of genomic, epigenomic, transcriptomic, and metagenomic analyses in bone genetic disorders.

## 1. Introduction

The world is now empowered by big data. In life science research, next-generation sequencing (NGS) has enabled the simultaneous reading of numerous fragmented DNA molecules and their sequencing in a massively parallel manner [1,2]. In 2003, the completion of the Human Genome Project was declared [3], and the Telomere-to-Telomere (T2T) consortium presented the complete sequence of the human genome, T2T-CHM13, in 2022 [4]. The release of all analyzed data has greatly expanded the research agenda. Using Sanger sequencing, the mainstream method at the time, the cost of analyzing an individual’s genome was approximately 10 USD million 20 years ago [5,6]. The promotion of the “Advanced Sequencing Technology Awards”, commonly known as the “1000 USD Genome Program”, has made it possible to bring the cost down to 1000 USD per person [7]. The ability to decode the human genome in a few days and its reduced cost has led many researchers to focus on using NGS to elucidate the genetic underpinnings of diseases [8]. Compared with conventional Sanger sequencing, NGS can yield an overwhelming amount of data in a short time [9].

Bone genetic disorder is a general term for diseases that cause abnormalities in the formation and maintenance of the skeleton because of defects in the growth, development, and differentiation of bone, cartilage, and other skeleton-forming tissues. Nosology and classification of genetic skeletal disorders 2023 revision for skeletal dysplasia lists 771 diseases, of which more than 90% are genetic diseases [10]. Many bone genetic diseases occur infrequently, but their causative genes are diverse. Although some causative genes have been elucidated, others remain unclear. However, investigating bone genetic diseases is valuable in that exploring the role of causative genes in human bone diseases will clarify their subsequent effect on developmental processes. Furthermore, the analysis of genetic mutations in many bone disorders will help develop new drugs and therapies, such as genome editing technology, for correcting mutated genes, especially in patients with genetic diseases that are difficult to treat [11,12].

NGS is indispensable for the analysis of bone genetic diseases. In this review, we discuss recent NGS analysis research in bone genetic disorders, showcasing its role in advancing our understanding and its applications.

## 2. Genome Database

In 2008, a research group at the University of Washington successfully sequenced the entire genomes of patients with leukemia and identified new candidate genes that may be related to the etiology of acute myeloid leukemia [13]. Since then, numerous studies have elucidated hereditary cancer development using germline and driver gene analyses by decoding the genome of cancer tissues [14,15]. In particular, The Cancer Genome Atlas (TCGA) was launched in the United States in 2006 [16,17], and the International Cancer Genome Consortium (ICGC) was launched in 2008 for large-scale cancer genome research on the 50 most important cancers and major subtypes worldwide [18,19]. The Pan-Cancer Analysis of Whole-Genome (PCAWG) project led by ICGC has integrated and analyzed whole-genome sequencing data for 2658 cases of 38 cancer types. In addition, St. Jude Children’s Research Hospital and the University of Washington School of Medicine in the U.S. plan on conducting a comprehensive analysis and sequencing the genomes of all pediatric cancer patients (https://www.stjude.org/research/translational-innovation/pediatric-cancer-genome-project.html) (accessed on 1 August 2023). Each patient has a different genome sequence, and by decoding the diversity of genome sequences, it is possible to make an appropriate diagnosis for each patient, and selecting treatment methods and drugs tailored to each patient is expected to yield effective results [20]. Furthermore, genetic data can be accumulated by decoding the genome sequences of all hospitalized patients, which is expected to be effective for future treatment and the elucidation of the causes of diseases.

Genome databases, including GeneBank of the National Center for Biotechnology Information (NCBI) in the United States and the Ensembl gene of the European Molecular Biology Laboratory (EMBL) operated by the European Bioinformatics Institute (EBI) in Europe, have been established [21]. The UCSC Genome Browser, developed and maintained by the University of California, Santa Cruz, is a public genomic information resource that is widely used in bioinformatics and genetics research. Genetic information is personal information, and ethical issues and personal handling must be strictly determined. However, the accumulation of genetic and decoded genomic information is necessary for the development of medicine.

## 3. Single Nucleotide Polymorphism and Genome-Wide Association Study

**Figure 1:**
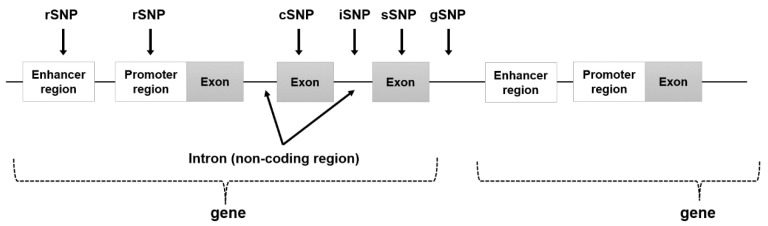
Location of each SNP in the gene region.

In the human genome, differences exist in the genomic sequences of individuals. A genomic sequence prevalent at a frequency of more than 1% of the population is called a genetic polymorphism. In contrast, at a frequency of less than 1%, it is considered a genetic variant [22]. Genetic polymorphisms are useful for determining disease-related risks, diagnosis, and drug efficacy. Single nucleotide polymorphisms (SNPs) are used as marker factors to search for disease-related genes [23,24]. SNPs are common polymorphisms in the human genome, occurring at a rate of approximately 1 per 1000 bases. Many SNPs are located in regions not involved in gene expression, whereas some occur in gene regions and are recognized as individual genetic differences that affect phenotypes. Regulatory SNPs (rSNPs) are expressed in the promoter and enhancer regions of genes and alter gene expression, while coding SNPs (cSNPs), present in exonic regions, result in amino acid substitutions and may be factors in disease susceptibility genes [25,26,27,28]. Other types of SNPs include untranslated SNPs (uSNPs) in non-coding regions within genes, introns SNPs (iSNPs) in intron regions, silent SNPs (sSNPs) in exon regions with single nucleotide substitutions that do not cause amino acid substitutions, and genomic SNPs (gSNPs) that exist outside the expressed gene. (Figure 1 and Table 1).

Visual representation of the location of each SNP in the gene regions shown in Table 1.

A variety of DNA microarrays have been developed to detect SNPs in specific genes. However, the sequence of the gene to be analyzed must be clear in advance. As a large number of genes are analyzed at the same time, false positives are common, and problems with quantitation are known to arise. Moreover, a few genetic diseases can be explained by a single or a small number of SNPs. However, in most cases, many genetic variants occur simultaneously and influence the disease phenotype. Therefore, searching and unraveling specific portions of the genome is insufficient, and the entire genome must be searched to identify mutations. Genome-wide Association Study (GWAS) analysis is now widely used to analyze the whole human genome.

Genome-wide Association Study searches for genetic polymorphisms associated with various human diseases and genetic variants that affect phenotypic diversity [29]. In 2002, the results of GWAS-based analyses were first reported worldwide. RIKEN reported SNPs associated with myocardial infarction through a massive analysis of hundreds of thousands to millions of data points worldwide [30]. This marked the beginning of SNP analyses using GWAS and has enabled the search for SNP allele frequencies and whole-genome sequencing (WGS) rare variants, as well as international collaborations to integrate SNP data. The International HapMap and 1000 Genomes Project aimed to build a haplotype map of human disease genomes [31,32]. These international projects have made it possible to analyze the genetic diversity of many DNA samples. In addition, GWAS results are publicly shared on the NHGRI-EBI GWAS catalog site (https://www.ebi.ac.uk/gwas/) (accessed on 1 August 2023), where GWAS play a significant role in elucidating genetic variation related to disease and in revealing the genetic architecture found in specific ethnic and racial populations.

**Figure 2:**
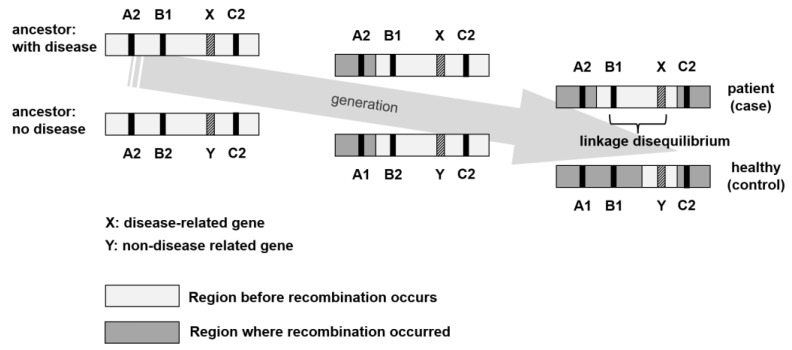
Example of linkage disequilibrium.

GWAS is a mapping method that uses representative SNPs covering the entire human genome as markers. It exploits linkage disequilibrium (LD) between the causative genetic variants for a phenotype and nearby SNP markers to identify the positions of genetic loci. Genes associated with diseases are called susceptibility genes and are believed to be present in the vicinity of SNPs. In other words, SNPs are present in isolation but may also be present in specific combinatorial states detected at high frequencies and may move together during crossing-over; this is called LD [33,34]. When two polymorphisms are found on the same chromosome in a population, they are considered to be in a linkage relationship, or LD. In a polymorphism in a linkage relationship, the history of recombination between the two polymorphisms from generation to generation is considered a characteristic of the population and not of the individual. A specific combination of these genes is essential for the identification of disease susceptibility genes. Therefore, by uncovering SNPs linked to specific diseases and identifying the SNPs in their vicinity, it is possible to identify critical genes that are key to treating these diseases (Figure 2).

In this case, the disease is assumed to be caused by the presence of the related disease gene X on the same chromosome as B1. In these patients, X and B1 are retained on the same chromosome as in the ancestor and have not undergone recombination. This relationship between X and B1 is called “chain disequilibrium”.

GWAS can comprehensively analyze the human genome by successfully incorporating LD. It also has fewer false positives than the SNP analysis, which has led to many studies. Urano et al. reported SNPs associated with low bone mineral density (BMD), osteoporosis, and osteoporotic fractures that have been identified over the past 20 years. These SNPs have been mapped close to genes encoding nuclear receptors and WNT-β-catenin signaling proteins [35]. Furthermore, BMD is genetically controlled by twin and familial studies.

GWAS can identify susceptibility loci but cannot determine how mutations affect a person, how they appear in the phenotype, or how they function. Therefore, NGS, described below, in combination with GWAS, can be used to analyze the functions of gene mutations.

## 4. Next-Generation Sequencing

**Figure 3:**
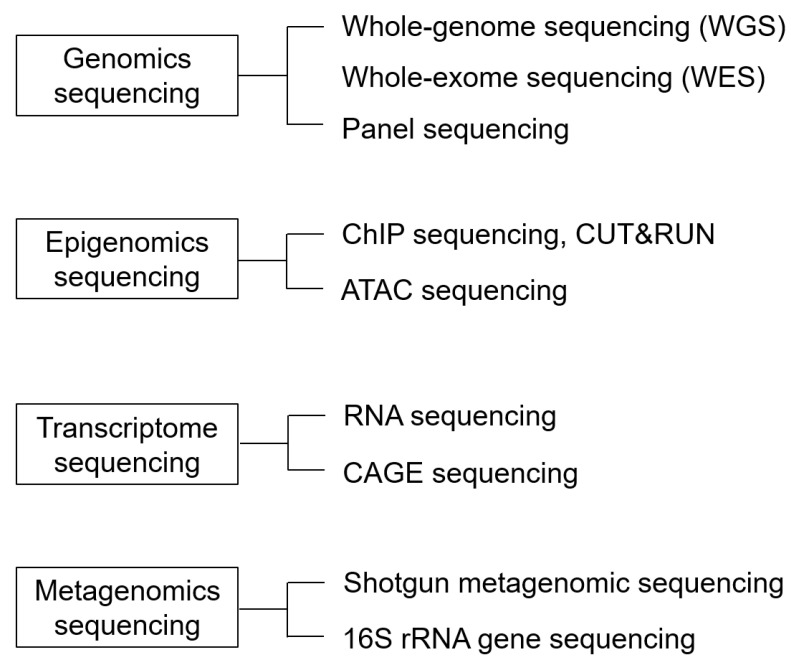
Various NGS-based sequencing methods.

The human genome has approximately 3.05 billion base pairs, and several methods can be employed for decoding them. The analysis method can be selected according to the purpose. Genomic analysis is one method used to decode the human genome. Epigenomic analysis deals with genetic information defined by chemical modifications of DNA or histone proteins without any changes in the DNA sequence. Transcriptome analysis comprehensively analyzes transcripts such as RNA transcribed from DNA [36] (Figure 3). We will give an overview of the research methods that have contributed significantly to the development of genetic research, along with the recent findings.

We categorized four major methods. The first is genomic sequencing, which comprehensively detects genomic mutations. The second is epigenomic sequencing, which examines the mode of genomic modifications involved in the regulation of gene expression, including DNA and histone modifications, chromatin conformation, and non-coding RNAs. The third is transcriptome sequencing, which comprehensively examines transcripts, and the fourth is metagenomic sequencing, which comprehensively sequences the DNA of all bacterial flora.

### 4.1. Genomics Analysis

**Figure 4:**
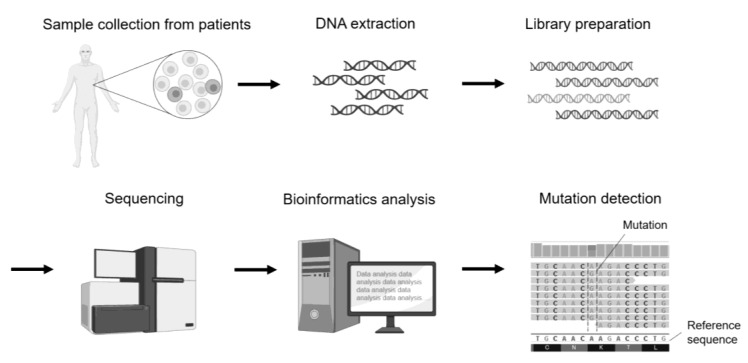
Schematic diagram of mutation analysis by genomics sequencing using a sample derived from a patient with a genetic disease.

Genomic analysis involves a comprehensive analysis of the human genome to determine the DNA sequences that make up the genome. Next-generation sequencers are commonly used to decipher the human genome, particularly disease-specific genomes. The primary purpose of genomic data analysis is to identify the genetic variants that cause the disease phenotype, including bone genetic disorders, from sequence data (Figure 4).

A sample is collected from the blood or other tissues of patients with genetic diseases, and DNA is isolated. The DNA is used to prepare a library, which is then sequenced using a next-generation sequencer. Bioinformatics analysis is performed on the obtained sequence data, and mutations are identified by comparing them with reference sequences.

#### 4.1.1. Advances in Genetic Analysis: From Gene Chips and Panels to Whole-Exome Sequencing (WES), and Finally, WGS

Even if a disease is assumed to be genetic, it is difficult to analyze genetic diseases with multiple causative genes using conventional Sanger sequencing, making the analysis more complex.

In general, the identification of a causative gene does not immediately lead to the elucidation of disease pathogenesis [37]. In some cases, various genetic abnormalities share a common pathology, such as muscular dystrophy. In the “Development of Human Muscle DNA Chip” all known cDNA information expressed in human skeletal and cardiac muscle was collected, and cDNA fragments of human skeletal and cardiac muscle were cloned in silico to eliminate cross-hybridization. The analysis of differences in gene expression between muscle diseases, the correlation between patients, and the signal transduction system analysis are considered promising in providing basic information for drug discovery.

Handling large amounts of raw data is difficult. Data collection over time and the identification of markers to evaluate the process of regeneration at the gene expression level will provide basic data for the future. The classification of related genes by cluster analysis may enable further pathophysiological analyses.

Patients with osteogenesis imperfecta (OI), a genetic disorder that causes repeated fractures, are clinically and genetically diverse. Mutations in COL1A1 or COL1A2 are causative in approximately 85–90% of cases. Mutations in IFITM5 [38], SERPINF1 [39], CRTAP [40], LEPRE1 [41], P3H1 [42], PPIB [43], SERPINH1 [44], FKBP10 [45], SP7 [46], BMP1 [47], TMEM38B [48], WNT1 [49], CREB3l1 [50], SPARC [51], FAM46a [52], MBTPS2 [53], MESD [54], SEC24d [55], CCDC134 [56], P4HB [57], PLOD2 [58], PLS3 [59], and KDELR2 [60] have also been reported. All these mutations affect type I collagen quality and quantity. A gene panel is an excellent tool for comprehensively analyzing and elucidating these mutations and is already commercially available [61]. In addition, the elucidation of pathological conditions by genotype–phenotype analysis is very important, and the use of gene panels for OI is becoming common. However, panel production and the additional discovery of new causative genes require a new set of panels, which is also costly. However, some unexpected genes may have been overlooked. This disadvantage can be circumvented by whole-exome sequencing (WES) [62]. The exome is a large panel of genes (approximately 45 Mb) that is enriched with all exons of the 20,000 genes known to date. The advantage of using WES is its potential to identify novel gene–disease associations. Most current genetic disease-causing gene searches use WES. However, the exome has gaps; for example, it only partially covers most introns. Therefore, a clear disadvantage of WES is its inability to identify deep intronic variants that can lead to disease-causing splicing abnormalities. The all-in-one solution for assessing coding and non-coding variants as well as structural variants (mainly copy number variants) is WGS. Future genetic analyses will likely converge rapidly with WGS analysis.

#### 4.1.2. Panel Sequencing

Recently, cancer genome medicine has become available in cancer medicine. Cancer gene panel tests, which can examine multiple genes simultaneously, are expected to provide tailor-made genome therapies, such as selecting effective drugs through the genetic analysis of patients. Such targeted resequencing is not limited to cancer but is also effective in diagnosing genetic diseases [63]. Using PCR, libraries are constructed and sequenced for specific regions. Consequently, one can concentrate on individual genes and target specific regions in the genome. Many whole-genome analyses have accumulated information, and databases have been constructed. Several bone disease panels, such as the osteogenesis imperfecta, osteopetrosis sugar genetic, and skeletal disease panels, are commercially available. In 2021, Nakamura et al. developed a diagnostic gene panel for Gorlin syndrome caused by gain-of-function mutations in the hedgehog signaling pathway. The gene panel analyzed the *PTCH1*, *PTCH2*, *SUFU*, and *SMO* genes, all of which are associated with Gorlin syndrome. They reported the presence of simultaneous mutations in PTCH1 and PTCH2 in patients with Gorlin syndrome [64].

#### 4.1.3. Whole-Exome Sequencing (WES)

Exome analysis, which decodes only the exonic regions, about 1.5% of the entire human genome, makes it possible to identify causative genes and nucleotide variants of inherited diseases [65]. Narrowing down the regions to be sequenced can reduce the time required to analyze an individual’s genome sequence. The decoded genome sequence is mapped using software, and only exonic regions with deletions and other genomic variants that cause genetic disease are extracted from the mapped file. In 2018, WES and panel sequencing analyses identified potential causal variants in 411 patients with skeletal dysplasia (288 families). Sharing phenotypic and genotypic data from a large molecularly characterized skeletal dysplasia cohort is expected to improve the diagnosis of these patients in the target population [66].

#### 4.1.4. Whole-Genome Sequencing (WGS)

WGS is a comprehensive method that analyzes the entire genome. Genomic information is expected to be helpful in the diagnosis and identification of hereditary diseases, the characterization of somatic mutations caused by cancer, and the identification of effective drugs for diseases [67]. It is also optimal for identifying and establishing novel genome sequences. It is possible to obtain an overall picture of the genome using genomic analysis. Moreover, it searches for any mutations that might have been missed using a targeted approach. In this respect, WGS has significant advantages. In 2020, Andersson et al. conducted a genetic search using WGS to determine whether patients with osteogenesis imperfecta (OI) and mutations in COL1A1/A2 had other genetic variants that might affect tooth development. Several missense variants were found, suggesting that the additive effects of pathological variants in COL1A1, COL1A2, and CERB3L1 may be necessary for osteogenesis imperfecta [68].

### 4.2. Epigenomics Sequencing

Epigenetic analysis is essential for elucidating bone diseases. Epigenetics refers to the further modification of a gene. Humans are multicellular organisms comprising many differentiated cells with a single genome. Several mechanisms control the expression of cell-specific genes. Epigenetics are the mechanisms that regulate gene expression without changing the DNA sequence, including DNA methylation, histone modification (methylation, acetylation, phosphorylation, etc.), and chromatin remodeling [69]. Information on the epigenetics of bone cells is obtained from the ENCODE project (http://www.genome.gov/encode/) (accessed on 1 August 2023).

#### 4.2.1. ChIP-Sequencing

Chromatin immunoprecipitation with high-throughput sequencing (ChIP-seq), a combination of chromatin immunoprecipitation (ChIP) assays and sequencing, is a powerful method for identifying the DNA-binding sites of transcription factors and other proteins on a genome-wide scale. In histone modifications, acetylated histones, especially acetylation of K9 and K27 on histone H3 (H3K9ac, H3K27ac), lead to chromatin decondensation and readily allow transcription into RNA, while trimethylation on K9 and K27 of histone H3 (H3K9me3, H3K27me 3) inhibits transcription [70]. Histone modifications during osteoblast differentiation have been examined using Chip-seq. The epigenetic regulation of Runx2, a master regulator of bone differentiation, involves the enrichment of H3K4me3 and H3K27ac marks in the Runx2 P1 promoter region [71]. Ankylosing spondylitis (AS) is a rheumatic disease with pathological bone formation that causes bone ankylosis and deformity; Yu et al. reported a 31 upregulated gene (SNP-adjacent superfamily) highly implicated in promoting abnormal osteogenic differentiation by integration of SNP data from ChIP-seq, RNA-seq, and the NHGRI-EBI GWAS catalog [72].

#### 4.2.2. ATAC-Sequencing

ATAC-seq analyzes only the sequences of open chromatin regions, which are genomic regions that are likely to be transcribed into RNA [73]. In ATAC-seq, cellular activation leads to an increase in accessible chromatin regions, resulting in enhanced transcriptional activity. Active genes adopt an open chromatin state, facilitating accessibility to transcription factors and RNA polymerase and leading to upregulated gene expression. Consequently, transcriptional products, including RNA (mRNA), increase [74]. Unlike ChIP-seq, this method is versatile as it does not require specific antibodies [75]. In 2018, Liu et al. performed ATAC-seq using articular knee cartilage from patients with osteoarthritis (OA), a common joint disease, and integrated the analysis with previously reported RNA-seq data from patients with OA. They confirmed that the promoters and enhancers of genes involved in OA pathogenesis are altered. These results suggest that aberrant enhancer usage is associated with mesenchymal stem cell (MSC) differentiation and chondrogenesis in OA [76].

### 4.3. Transcriptome Sequencing

Transcriptome analysis is performed on mRNA and lncRNA as a post-sequencing analysis for genomic analysis, which determines the DNA sequence on a genome-wide basis. In 1998, Yasuda et al. reported that the receptor activator of nuclear factor-κB ligand (RANKL) is an important factor essential for osteoclast differentiation and activation [77]. The RANKL-RANK signaling pathway in osteoclasts was clarified, and further studies were conducted on the downstream transcriptional mechanism. In 2002, Takayanagi et al. used Affymetrix oligonucleotide arrays to analyze transcriptome changes over time after M-CSF and RANKL stimulation of bone marrow-derived macrophages. They reported that the NFATc1-dependent transcriptional program is continuously induced during RANKL osteoclast differentiation. In addition, because NFATc1-deficient embryonic stem cells do not respond to RANKL stimulation and do not differentiate into osteoclasts, and ectopic expression of NFATc1 allows progenitor cells to differentiate efficiently without RANKL signaling, NFATc1 functions downstream of RANKL and is a master regulator of osteoclast terminal differentiation. NFATc1 may be a master switch that functions downstream of RANKL to regulate the terminal differentiation of osteoclasts [78].

#### 4.3.1. RNA Sequencing

RNA-seq has become popular as an alternative to gene chips for the comprehensive analysis of gene expression [79,80]. Onodera et al. used RNA-seq to analyze the transcriptome of Gorlin iPS cell-derived osteogenic populations. They identified the upregulation of several transcription factors that are targets of hedgehog signaling and showed that these transcription factors may be involved in promoting osteoblastogenesis and calcification in Gorlin iPSCs [81]. In addition, using an integrated analysis of ATAC-seq, ChIP-seq, and RNA-seq, Hojo et al. showed that chromatin accessibility differed between osteoblasts and chondrocytes in neonatal mice. Furthermore, they found that the Sp7 distal enhancer driven by Runx2 contributes to osteoblast differentiation [82].

#### 4.3.2. CAGE Sequencing

Cap analysis of gene expression-seq (CAGE-seq) is a method developed at RIKEN that allows quantitative analysis of each gene at each transcription start site and genome-wide promoter activity. It is a comprehensive method for analyzing mRNA and lncRNA expression levels at transcription start sites [83].

As a new transcriptome analysis method, CAGE-seq, which can analyze gene expression and regulatory regions, has been reported to reveal diversity in gene expression regulation [84]. In contrast to RNA-seq for full-length analysis, CAGE-seq utilizes a 5’ end cap structure and determines the sequence at the 5’ end of the transcript. The determined sequence is compared to the genomic sequence to identify the transcription start site on a genome-wide basis [85]. The authors used CAGE-seq to perform total transcriptional profiling of induced pluripotent stem (iPS) cells (CCDiPS) derived from patients with cleidocranial dysplasia (CCD) with a genetic mutation in *RUNX2* after osteoblast induction. This analysis revealed that the *RUNX2* promoter P2, but not the *RUNX2* P1 promoter normally used in bone tissue, was activated in iPS cell-derived osteoblasts. We also identified a novel ncRNA involved in osteoblast differentiation [86].

### 4.4. Metagenomic Sequencing

Metagenomic analyses, in which next-generation sequencers decode the DNA sequences of bacterial flora, have also been developed. The shotgun method extracts and collects the genomic DNA of all microorganisms in an environment without culture, comprehensively deciphers these sequences, deciphers the 16S rRNA sequence, and analyzes the proportion of various bacteria present [87]. These methods allow us to identify the presence of microorganisms [88]. Ye et al. reported that the pathogen detection rate of mNGS in the dialysis effluent of peritonitis patients was significantly higher than that of traditional cultures. Metagenomic analysis is particularly suitable for oral microflora studies [89].

The oral cavity is a tissue composed of two surfaces, the mucosa and teeth, and this complex structure is helpful for microbial colony formation. More than 200 bacteria have been detected in the human oral cavity [90,91]. Wang et al. collected dental swabs or dental plaque from healthy humans and patients with chronic periodontitis and used metagenomic sequencing for a comprehensive review of the microbial communities in both [92]. Thus, elucidating the characteristic genome of the complex structure of the oral microbiota will lead to a better understanding of alveolar bone destruction.

## 5. Single-Cell Analysis

Single-cell RNA sequencing (scRNA-seq) has recently enabled the isolation and analysis of single cells. Conventional cell analysis methods analyze cellular processes such as cell metabolism, motility, differentiation, and proliferation on a population basis. However, it is difficult to analyze cellular heterogeneity because it uses the average value of a cell population [93,94]. However, scRNA-seq can detect changes in cell abundance, identity, and gene expression in skeletal cells. In particular, the analysis of the transcripts of a cell population on a cell-by-cell basis has advantages, for example, in extracting specific gene expression and trends of bone genetic disorders on a cell-by-cell basis after the induction of differentiation. Because scRNA-seq can be performed on a cell-by-cell basis, it is effective in elucidating many genetic diseases. It can be combined with conventional RNA-seq, ChIPseq, and ATACseq to perform exhaustive analyses [95,96].

## 6. Conclusions

NGS has advanced life sciences to new heights. The development of genome analysis technologies has enabled the elucidation of the genetic underpinnings of various pathological conditions. NGS is expected to be particularly useful in identifying individual genetic variability for diagnosing genetic disorders and offering hope to patients. Research on NGS for bone lineage genetic diseases is still in its infancy. Unlike the analysis, diagnosis, and treatment of cancer genomes, which focus on localized areas, establishing genomic therapies is essential for genetic diseases. Advancements in human genome analysis and the use of NGS are expected to lead to further improvements in therapeutic techniques and the establishment of personalized treatment approaches.

## Figures and Tables

**Table 1:** Features of each SNP by location.

Classification	Location	Features
rSNP (regulatory SNP)	enhancer and promoter region (regulatory region)	Alters gene expression and may alter phenotype.
cSNP (cording SNP)	exon region (translation region)	Change the type of amino acid (missense or nonsense mutation). Nonsense mutations change the phenotype, while missense mutations may or may not change the phenotype.
sSNP (silent SNP)	exon region (translation region)	Because it is a silent mutation with no change in amino acid type, the possibility of phenotypic change is extremely low.
iSNP (intron SNP)	intron region (regulatory region)	Alters gene expression and may alter phenotype.
gSNP (genomics SNP)	junk region	Little or no effect on gene expression levels and phenotypic changes.

## Data Availability

Not applicable.
